# Dried Black Soldier Fly (*Hermetia illucens*) Larvae in a Sustainable Diet for Laying Hens: Effects on Welfare and Behavior

**DOI:** 10.3390/ani16111724

**Published:** 2026-06-04

**Authors:** Yosra Znazen, Marwa Gaddes, Geert P. J. Janssens, Madiha Hadj Ayed

**Affiliations:** 1 Institut Supérieur Agronomique de Chott-Mariem, Université de Sousse, LR18AG01, ISA-CM-BP, 47, Sousse 4042, Tunisia; gaddesmarwa@gmail.com (M.G.); mediha.ayed@yahoo.fr (M.H.A.); 2 Department of Veterinary and Biosciences, Ghent University, Heidestraat 19, B-9820 Merelbeke, Belgium; geert.janssens@ugent.be

**Keywords:** black soldier fly, laying hens, body scoring, larvae consumption time, novel object

## Abstract

This study evaluated the effects of locally sourced ingredient dietary, with or without supplementation of black soldier fly (*Hermetia illucens*; BSF) larvae, on laying hen welfare. A total of 150 Lohman White hens aged 30 weeks were assigned to three treatments over ten weeks: a standard corn–soybean diet (CONTROL), an alternative diet incorporating triticale, faba beans and rapeseed meal (ALTER), and the ALTER diet supplemented with 5% dried BSF larvae provided separately (ALTER + BSF). Welfare assessments included larvae consumption time, a novel object test, an avoidance distance test, body condition scoring, and ethological observation of natural behaviors. Hens fed ALTER diet initially showed increased incidence of comb pecking wounds, which declined over the trial, along with reduced morning grooming compared to the CONTROL group (*p* = 0.009). However, the ALTER diet significantly improved plumage cleanliness (*p* < 0.001). Supplementation with BSF larvae partially mitigated early stress responses, maintained plumage cleanliness, and improved exploratory behavior and habituation to novelty (*p* < 0.001). Hens showed sustained and increased motivation to consume BSF larvae with an average consumption time of 5.5 min. Additionally, BSF supplementation was associated with increased resting and the emergence of dustbathing behavior during the afternoon (*p* < 0.05). No aggressive behaviors were observed, and no significant dietary effects were found for human fearfulness throughout the trial. In conclusion, dried BSF larvae can serve as effective environmental enrichment, improving hens’ adaptability to locally sourced diets in rural farming systems.

## Simple Summary

The adoption of sustainable feed ingredients, such as locally available feedstuffs and insects, constitutes a promising and cost-effective option for smallholder farmers, underscoring the importance of evaluating their implications for animal welfare. In this study, a total of 150 hens were given either a standard diet based on imported ingredients, a diet made from local ingredients, or the same local diet topped with dried black soldier fly (BSF) larvae. Birds’ welfare and behavioral indicators were assessed over ten weeks. Initially, hens fed the local diet showed some signs of stress, such as more pecking and less grooming, which declined over time with improved feather cleanliness. Hens fed locally sourced diet supplemented with BSF showed increasing interest in BSF larvae over time. They explored more, became less fearful of new objects, and showed higher positive behaviors like resting and dustbathing. No aggressive behavior or increased fear of humans was observed in any group. Overall, this study suggests that environment enrichment with dried black soldier fly larvae can be a useful strategy, helping hens to improve welfare when using locally sourced feeds.

## 1. Introduction

Advances in genetics, nutrition, and husbandry systems have primarily focused on optimizing hen productivity, feed efficiency, and the cost-effective production of high-quality eggs. However, feeding a complete diet formulated solely to meet production requirements has often occurred at the expense of opportunities for natural behaviors, thereby raising societal and scientific concerns regarding laying hen welfare in poultry production systems, including rural and small-scale farming contexts.

Nutrition and feed management play a central role in animal health and welfare [1]. In Tunisia, as a case, the inclusion of locally produced ingredients in poultry diets has emerged as a sustainable strategy to improve feed self-efficiency for small farmers. For example, triticale has been widely cultivated as a suitable source of energy (13.5 MJ/kg) [2,3]. Faba beans emerged as a substitute for soybean meal, particularly high in lysine, which supports growth, immune function, and feather development, thereby contributing to stronger muscle formation and improved plumage [4,5]. Rapeseed meal, when included at recommended levels, can enhance the supply of sulfur amino acids (methionine and cysteine) in the diet, which plays a crucial role in egg formation [6]. Therefore, the incorporation of those ingredients in laying hens’ diet may modify key dietary characteristics, notably fiber content, which may influence feeding behavior and stress responses in laying hens [7,8,9].

Furthermore, providing opportunities for birds to express their natural behavior has become a key component of welfare-oriented production. Environmental enrichment through black soldier fly (*Hermetia illucens*; BSF) larvae serves as an effective practice for rural and smallholder farmers, simultaneously addressing the nutritional needs of the birds and improving their welfare [10,11]. Indeed, a growing body of evidence confirms that live BSF larvae positively influence activity levels and welfare-related behaviors in poultry [12,13,14,15,16]. However, their practical deployment in smallholder farms is often hindered by storage, hygiene, and nutritional volatility, factors that limit their scalability and reliability in small-scale farming systems.

Dehydrated forms offer a more practical alternative, providing extended shelf life, stable nutritional composition, easier handling, and improved biosecurity. Early evidence suggests that chickens readily accept dried BSF and that its supplementation promotes natural foraging behavior, improves litter quality, and reduces harmful behaviors such as feather pecking [11,17,18]. Yet despite these promising findings, the welfare implications of the dried BSF remain subsequently underexplored, particularly when used as supplement alongside locally sourced feed ingredients.

Therefore, this study aimed to assess the welfare implications of partially substituting corn and soybean meal with locally sourced feed ingredients in laying hens, with and without whole dried BSF larvae as environmental enrichment.

## 2. Materials and Methods

### 2.1. Birds, Housing, and Diets

All procedures involving animals in this study were approved by the Animal Ethics Committee of the National School of Veterinary Medicine, Sidi Thabet (Tunisia). A total of 150 Lohmann White laying hens were acquired from a commercial breeding farm at 28 weeks of age. The hens were assigned to 15 enclosed group pens (1.9 m × 1.12 m × 6 m H × W × L), providing a stocking density of 0.67 m^2^ per bird, within a floor-based housing system with semi-open design. Each pen was equipped with collective nest boxes and perches offering 168 cm2 of nest space and 22.5 cm of perch per hen. A 10 L drinker, a suspended circular feeder and a professional 8 MP (4K) camera (Hikvision DS-2CD2T83G0-I8, Hangzhou Hikvision Digital Technology Co., Ltd., Hangzhou, China) were installed. The covered internal area, representing two-thirds of the pen’s surface, was lined with wood shavings, with fresh layers added weekly. Hens were allowed a two-week adaptation period with ad libitum access to feed and water under a 16 h light:8 h dark photo period. The experimental period was conducted from March to June, during which the ambient temperature ranged from 14.5 °C to 33 °C. At 30 weeks of age, cages were randomly assigned to one of three dietary treatments (5 replicates per diet):

The first group (CONTROL) received a conventional pelleted corn–soybean meal diet (11.44 MJ/kg metabolizable energy, 17.01% crude protein). The second group (ALTER) received a pelleted, locally sourced diet (ALTER) incorporating 20% triticale, 10% faba beans and 5% rapeseed meal (11.44 MJ/kg metabolizable energy, 17.07% crude protein). The third group (ALTER + BSF) consisted of the ALTER diet supplemented with whole dried BSF larvae, adjusted to 5% of the expected daily dry matter intake, and administered separately from the main feeder in dedicated plates. The 5% supplementation level was adopted based on levels reported in the closest available literature on dried BSF larvae in poultry welfare studies, namely 5% in Fiorilla et al. [17] and 8% in Ipema et al. [11], both reporting measurable welfare studies. Given the scarcity of welfare-focused studies using dried BSF in laying hens, 5% was considered a biologically relevant and conservative starting point. However, the BSF larvae used in this study are composed of 5.7% moisture, 43.6% crude protein, 30.54% crude fat, 8.3% crude fiber, 2.34% chitin and an estimated metabolizable energy content of 17.79 MJ/kg on a dry matter basis. ALTER + BSF diet had a calculated metabolizable energy content of about 11.75 MJ/kg and crude protein content of about 17.35% on a dry matter basis.

The formulation and nutrient composition of the basal diets are presented in Table 1. Birds had *ad libitum* access to water and feeds during the 10 weeks of trial. Pelleted feeds were distributed daily at 9 am. The same operator performed in-cage tasks such as daily feed and water provision, BSF larvae distribution and welfare tests over the trial.

### 2.2. Larvae Eating Time

Throughout the ten weeks of trial, ALTER + BSF pens received a daily portion of dried BSF larvae separately from feeds, distributed in two plates at 11 a.m. To maintain consistent interaction with the operator across all treatments, two empty plates were placed in CONTROL and ALTER pens. The larvae consumption time was daily recorded using a stopwatch, starting from the moment the operator placed the plates inside the cages [14]. The remaining quantity of BSF larvae left after 30 min of recording time was added to the pelleted concentrate into feeders.

### 2.3. Novel Object Test

To evaluate changes in fear-related behavior and habituation responses to a novel object, the laying hens were observed on four occasions throughout the trial (days 16, 33, 48, and 62 of the trial) in the afternoon during their exposure to an object consisting of a 20 cm stick covered with colored stripes (blue, yellow, and red). During each test, the operator placed the object at a standardized location. Thirty seconds after leaving the pen, the number of hens approaching the novel object within a radius of one hen’s body length (15 cm) was recorded every 10 s over a duration of two minutes [19,20].

### 2.4. Avoiding Distance Test

The avoidance distance test was conducted four times during the experiment on days 10, 30, 47, and 63 to evaluate the effect of diet on fearful responses of the hens toward humans. In the afternoon, the operator entered the inner part of the pen, approached the hens, squatted on the litter for 10 s, and then counted the number of hens approaching within an arm’s length (approximately 1 m). This procedure was conducted at designated observation points, which were similar across all the pens [21].

### 2.5. Body Scoring

All birds were subjected to body scoring assessment following the welfare quality assessment protocol for laying hens [22]. This test was conducted three times during the trial: at the beginning (day 1), middle (day 37), and the end of the experiment (day 70). For each hen, body condition was evaluated through the examination of plumage cleanliness, skin lesions, hock burns, footpad dermatitis, plumage damage in the back of the head, comb pecking wounds, and feather condition for back, neck, rump, and belly. For each parameter, scores were assigned according to the criteria described in Table 2.

### 2.6. Animal Behavior Assessment

Behavioral observations were conducted on days 18, 42 and 69 of the trial to assess early, mid-trial and long-term responses to dietary treatments. Recordings were performed three times per day: in the morning, one hour before feed distribution (T0), during provision of BSF larvae (T1), and in the afternoon between 3 and 4 p.m. (T2). For each session, five minutes of video were analyzed with BORIS interactive Software, version 7.13.8, following the methods described by Friard et al. [23], using a standardized ethogram (Table 3). All videos were treated by an observer blind to the treatment group. Pecking behaviors, along with foraging and wing flapping, were recorded as point events and the remaining behaviors (drinking, eating, standing, sitting, walking, grooming, and dustbathing) were recorded as state events.

### 2.7. Statistical Analysis

Statistical analyses were performed using R software (version 4.4.0). Larvae consumption time and body scoring parameters were analyzed using a linear mixed-effects model (LMM) via the “lme4” package. For novel object and avoidance distance tests, data were analyzed using a generalized linear mixed model (GLMM) via the “glmmTMB” package. A standard binomial error distribution was used, including dietary treatment, test day, and their interaction as fixed effects, and pen as a random intercept to account for repeated measures. For the natural behavior assessment, data collected at the individual bird level were aggregated to the pen level, which served as the statistical unit. Behavioral durations were analyzed using LMM and behavioral frequencies were analyzed using GLMM with Tweedie distribution, handling the zero-inflated continuous data. Fixed effects included diet, observation day, time of day, and their two-way and three-way interactions, with pen as a random effect. The significance of the fixed effects was assigned via a type III ANOVA from the car package (Wald Chi-square (χ^2^) for GLMM; F-tests for LMM). Post hoc pairwise comparisons were conducted on the least square means (LSMeans) using the emmeans package, applying the Tukey method for multiple comparison adjustments (*p* < 0.05).

## 3. Results

### 3.1. Larvae Eating Time

**Figure 1:**
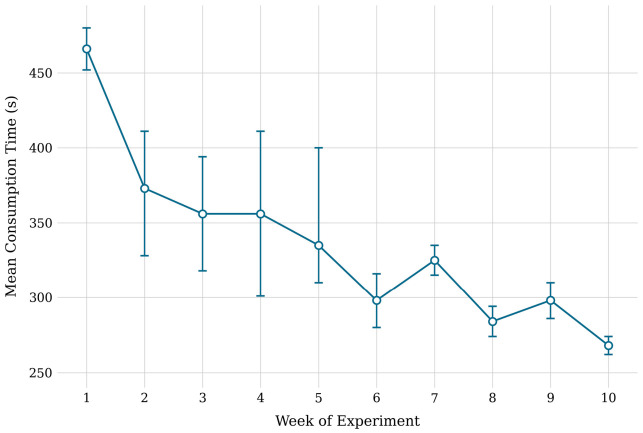
Larvae consumption time (means ± SE) recorded during ten weeks of trial of laying.

The time spent by the hens eating the entire daily portion of larvae (Figure 1) averaged 337 s, with a significant decrease observed over weeks (*p* < 0.001). In the first week, the laying hens spent longer time finishing their daily portion of BSF (465 s). Thereafter, consumption time converged to decrease proportionally over the weeks, showing an average of 266 s in the last week.

### 3.2. Novel Object Test

The responses of laying hens to the novel object test throughout the trial are presented in Table 4. The statistical model revealed a highly significant interaction between diet and day (*p* < 0.001), indicating that the response of laying hens to the object varied significantly between the dietary groups over time. On day 10, hens fed ALTER diet showed significantly lower interest in the novel object (24.8%) compared to the CONTROL group (35%), while the ALTER + BSF group showed an intermediate value (26.9%; *p* < 0.001). A shift occurred on the second observation, characterized by a decrease in the CONTROL group reactivity (18.7%), significantly lower than that in the ALTER and ALTER + BSF groups that showed similar reactivity (28% and 33% respectively). Thereafter, hens fed ALTER + BSF diet showed significantly the highest approaching rates compared to the CONTROL and ALTER groups, which maintained similarly low interaction values until the end of the trial. Overall, dietary treatment significantly affected hens’ interactions with the novel object (*p* < 0.001). ALTER + BSF exhibited the highest approaching frequencies (34.8%) compared to the CONTROL and ALTER diets, which were similar (22.7% and 26.45% respectively).

### 3.3. Avoidance Distance Test

**Figure 2:**
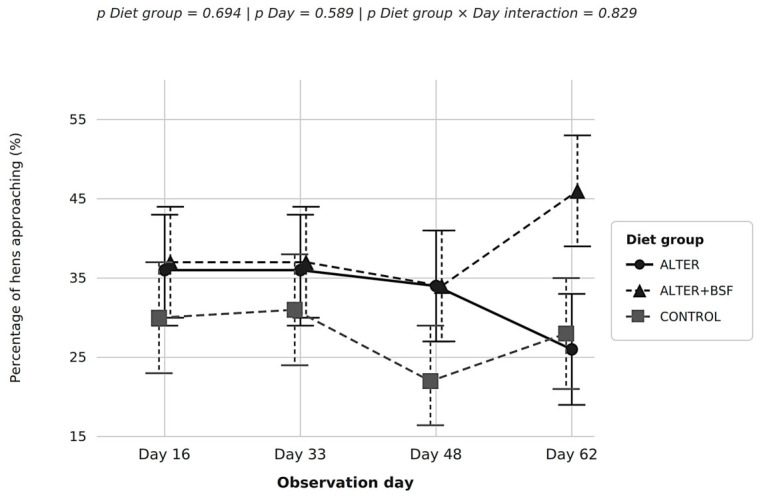
Percentage of birds (mean ± SE) approaching the operator during the four avoidance distance tests assessed in laying hens fed standard (CONTROL), alternative without (ALTER) or with black soldier fly (ALTER + BSF) diets (*n* = 5).

Avoidance distance test results (Figure 2) showed that dietary treatments did not affect the approaching percentage of the hens to humans over time (*p* = 0.82). Despite the tendency to increase observed in the ALTER + BSF group on the fourth day (46%), this observation did not reach significant levels (*p* > 0.05).

### 3.4. Body Scoring

**Figure 3:**
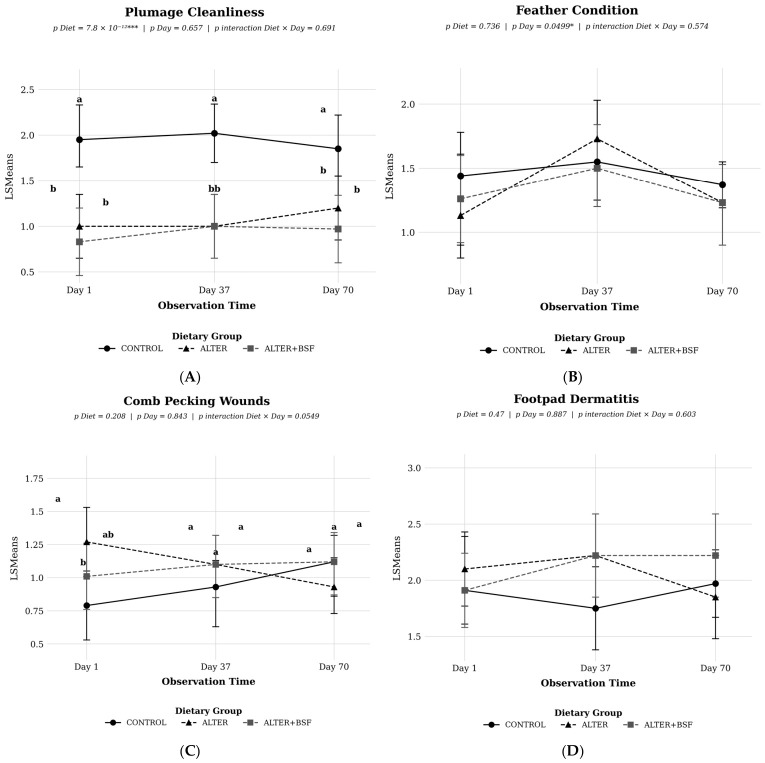
Body condition (LS means ± SE) examination of laying hens fed standard (CONTROL), alternative without (ALTER) or with black soldier fly (ALTER + BSF) diets observed in the beginning (day 1), middle (day 37) and at the end of the trial (day 70) (*n* = 5). (**A**) Plumage cleanliness, (**B**) feather condition, (**C**) comb pecking wounds, (**D**) footpad dermatitis. Different letters (a, b) indicate significant differences between the groups (*p* < 0.05) on the same observation day. * *p* < 0.05; *** *p* < 0.001.

The body scoring results, presented in Figure 3, showed that the CONTROL group consistently displayed higher LS means for plumage cleanliness (*p* < 0.05; Figure 3A), reflecting a high proportion of hens with dirty plumage (with a score of 2). In contrast, hens fed the ALTER and ALTER + BSF diets showed consistently lower LS means, indicating more hens with a plumage cleanliness score equal to 1, with small, localized spots of dirt or fecal material. Comb pecking wounds varied significantly at the start of the trial; the ALTER group displayed higher LS means, reflecting more cases of three pecking wounds or more, compared to the CONTROL group that showed the lowest levels, indicating more hens with less than 3 pecking wounds, while hens fed ALTER + BSF diet showed intermediate levels (Figure 3C). However, these scores converged in the middle and end of the trial, resulting in the absence of a significant dietary effect in the overall comb pecking wound scores (*p* = 0.2). Furthermore, no significant differences (*p* > 0.05) were observed among the three dietary treatments for feather condition (back, neck, rump, and belly; Figure 3B) or footpad dermatitis scores (Figure 3D). No signs of hock burn, plumage damage in the back of the head, or skin lesions were observed in any group throughout the trial.

### 3.5. Ethogram of Natural Behavior

**Figure 4:**
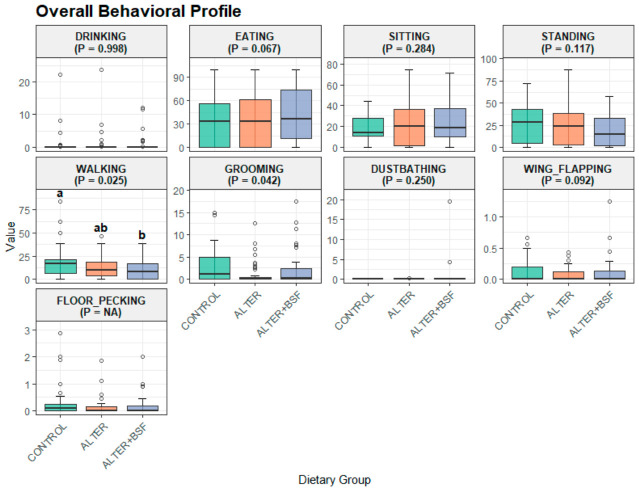
Global behavioral profile (LS means) of laying hens fed a standard (CONTROL), alternative without (ALTER) or with dried black soldier fly larvae supplementation (ALTER + BSF) diets. Duration behaviors (drinking, eating, sitting, standing, walking, grooming and dust bathing) are expressed as LS means of the percentage of observation time spent in each behavior at the pen level. Frequency behaviors (wing flapping and floor pecking) are expressed as LS means per pen (*n* = 5 pens per treatment). Boxplots represent the LS means (% duration or frequency), with the median indicated by the horizontal line, the box representing the interquartile range (25th–75th percentiles), and Whiskers extending to 1.5 times the interquartile range. Different letters (a, b) indicate significant differences between the groups (*p* < 0.05).

The global behavioral profile of the laying hens (Figure 4) revealed significant effects limited to walking and grooming. The ALTER + BSF group showed the lowest walking activity (9.62%) compared to the CONTROL group (17.82; *p* = 0.025). A significant effect of dietary treatment was found for grooming (*p* = 0.04), while pairwise comparisons did not reveal significant differences between specific groups.

**Figure 5:**
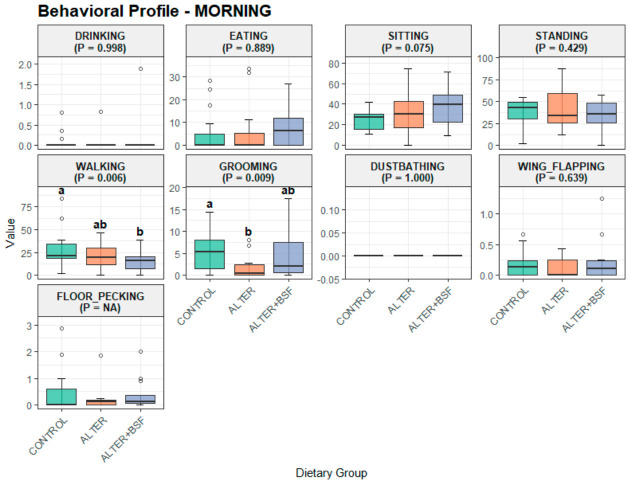
Behavioral profile (LS means) of laying hens fed a standard (CONTROL), alternative without (ALTER) or with dried black soldier fly larvae supplementation (ALTER + BSF) diets during pooled morning observations. Duration behaviors (drinking, eating, sitting, standing, walking, grooming and dust bathing) are expressed as LS means of the percentage of observation time spent in each behavior at the pen level. Frequency behaviors (wing flapping and floor pecking) are expressed as LS means per pen (*n* = 5 pens per treatment). Boxplots represent the LS means (% duration or frequency), with the median indicated by the horizontal line, the box representing the interquartile range (25th–75th percentiles), and Whiskers extending to 1.5 times the interquartile range. Different letters (a, b) indicate significant differences between the groups (*p* < 0.05).

Analysis of pooled morning observations (Figure 5) revealed that the ALTER + BSF group exhibited significantly decreased walking activity to half (14.6%) compared to the CONTROL group (28.84%), while the ALTER group showed intermediate values (20.23; *p* = 0.006). Crucially, grooming activity significantly decreased in the ALTER group (1.62%) compared to the CONTROL group (5.16%; *p* = 0.009). However, feeding ALTER + BSF diet restored grooming values to levels similar to those in the CONTROL group (4.24%). No significant differences were observed for the remaining behaviors between the groups during this time (*p* > 0.05).

**Figure 6:**
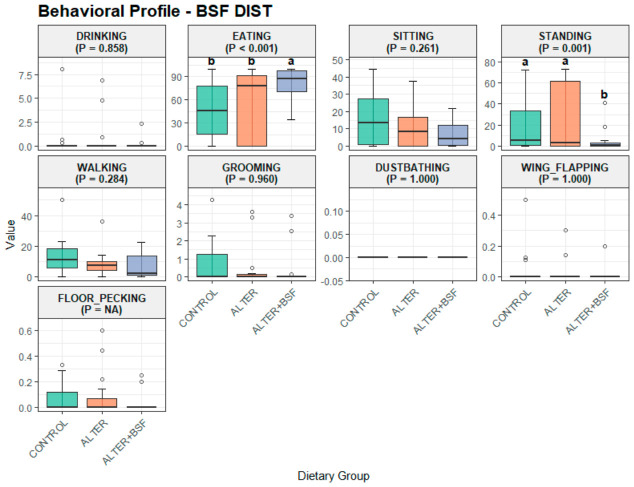
Behavioral profile (LS means) of laying hens fed a standard (CONTROL), alternative without (ALTER) or with dried black soldier fly (BSF) larvae supplementation (ALTER + BSF) diets during pooled larvae distribution time observations. Duration behaviors (drinking, eating, sitting, standing, walking, grooming and dust bathing) are expressed as LS means of the percentage of observation time spent in each behavior at the pen level. Frequency behaviors (wing flapping and floor pecking) are expressed as LS means per pen (*n* = 5 pens per treatment). Boxplots represent the LS means (% duration or frequency), with the median indicated by the horizontal line, the box representing the interquartile range (25th–75th percentiles), and Whiskers extending to 1.5 times the interquartile range. Different letters (a, b) indicate significant differences between the groups (*p* < 0.05).

Analyses of pooled observations of dried BSF larvae distribution time (Figure 6) revealed that eating activity was the dominant behavior in the ALTER + BSF group (81.2%), significantly higher than that in the CONTROL and ALTER groups (*p* > 0.001), which exhibited similarly lower durations (50 and 54.33% respectively). In contrast, hens in the CONTROL and ALTER groups similarly spent the majority of their time standing (20.36 and 24.72% respectively), significantly higher than that in the ALTER + BSF group (*p* = 0.001). No significant differences were observed for the remaining behaviors between the groups during this time (*p* > 0.05).

**Figure 7:**
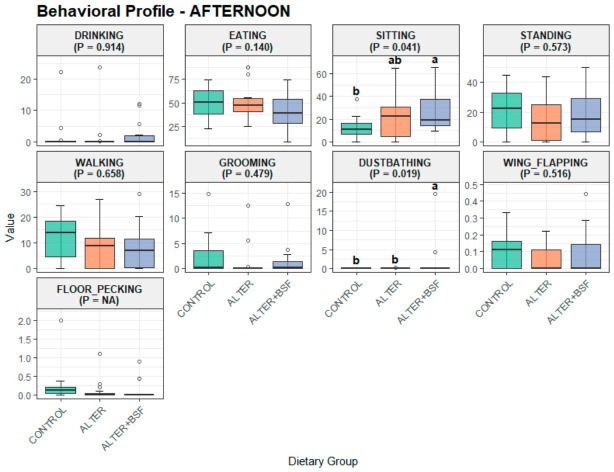
Behavioral profile (LS means) of laying hens fed a standard (CONTROL), alternative without (ALTER) or with dried black soldier fly (BSF) larvae supplementation (ALTER + BSF) diets during pooled afternoon observations. Duration behaviors (drinking, eating, sitting, standing, walking, grooming and dust bathing) are expressed as LS means of the percentage of observation time spent in each behavior at the pen level. Frequency behaviors (wing flapping and floor pecking) are expressed as LS means per pen (*n* = 5 pens per treatment). Boxplots represent the LS means (% duration or frequency), with the median indicated by the horizontal line, the box representing the interquartile range (25th–75th percentiles), and Whiskers extending to 1.5 times the interquartile range. Different letters (a, b) indicate significant differences between the groups (*p* < 0.05).

Data of pooled afternoon observation (Figure 7) showed that hens fed ALTER + BSF diet spent more time sitting (26.7%) compared to the CONTROL group (12.31%; *p* = 0.041), while the ALTER group showed intermediate values (21.45%). Furthermore, the ALTER + BSF diet significantly increased time spent in dustbathing (1.6%) compared to the CONTROL and ALTER groups, where this behavior was observed rarely (*p* = 0.019). No significant differences were observed for the remaining behaviors between the groups during this time (*p* > 0.05).

**Figure 8:**
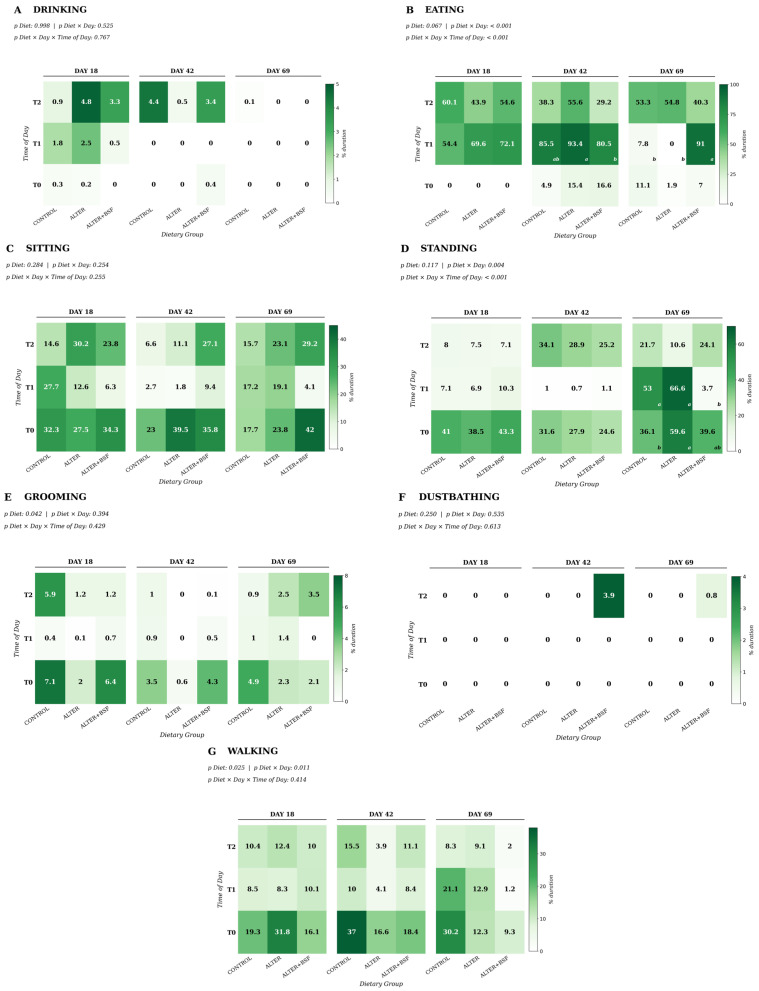
Heatmap distribution of behavioral time percentage (LS means) in laying hens fed standard (CONTROL), alternative without (ALTER) or with black soldier fly (ALTER + BSF) diets. (**A**) Drinking, (**B**) eating, (**C**) sitting, (**D**) standing, (**E**) grooming, (**F**) dustbathing, (**G**) walking. T0: morning, T1: black soldier fly distribution, T2: afternoon. Different letters (a, b) indicate significant differences between the groups (*p* < 0.05). Different letters (a, b) indicate significant differences between the groups (*p* < 0.05).

**Figure 9:**
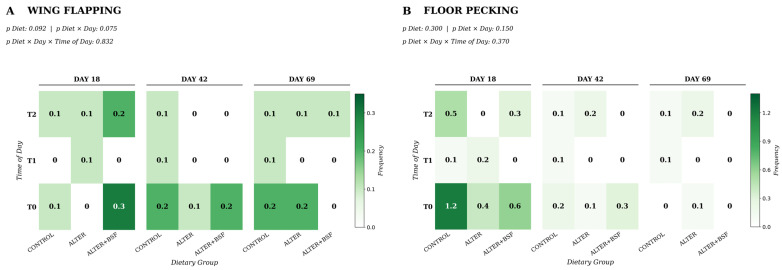
Heatmap distribution of point event frequencies (LS means) in laying hens fed standard (CONTROL), alternative without (ALTER) or with black soldier fly supplementation (ALTER + BSF) diets. (**A**) Wing flapping, (**B**) floor pecking. T0: morning, T1: black soldier fly distribution, T2: afternoon.

A significant three-way interaction (Diet × Day × Time) was observed only for eating and standing behaviors (Figure 8; *p* < 0.001). In the afternoon of day 42, a significant effect of diet emerged when the ALTER + BSF group showed reduced eating duration (29.2%) compared to the ALTER group (55.6%), while the CONTROL group showed an intermediate value (38.3%). However, by day 69, the ALTER + BSF group maintained a high eating frequency (90.96%; *p* < 0.001) and low standing value (3.75%; *p* < 0.001), while the ALTER and CONTROL groups reduced their eating activity (7% and 0% respectively, *p* > 0.05) and spent more time standing (53 and 66.5% respectively; *p* > 0.05) during BSF larvae distribution. Dustbathing behavior was not detected at the beginning of the trial; then, it emerged in the afternoon of the middle and end-trial observations. Pecking other birds (gentle, severe, or aggressive) and foraging behaviors were not detected across the three observation days. Wing flapping and pecking floor events rarely occurred (Figure 9).

## 4. Discussion

Larvae consumption time result shows successful acceptance and adaptation to the BSF larvae. The initial slow consumption reflects caution rather than a lack of hunger, likely attributed to the neophobic nature in laying hens that persists despite domestication [27]. The decrease in consumption time demonstrates that habituation occurred over time. While most studies on BSF feeding behavior measure the consumption time of live larvae, fewer have focused on the dried form. In fact, the wiggling movement of live larvae may visually trigger an immediate, innate predatory reflex [28]. Dabbou et al. [29] found that laying hens required 5 to 6 min to consume live BSF larvae (10–20% inclusion). Similarly, Bellezza Oddon et al. [15] reported 2.5 to 3.3 min for a 6% inclusion, exhibiting an adaptation pattern over time, similar to our findings. Contrarily to this quick reflex, consumption of immobile dried larvae is likely a longer learning path that triggers exploratory behavior, primarily driven by strong odor-active compounds and high density of glutamic acid and lipids [30,31], which may increase palatability and memorize the BSF dried larvae as a high-value nutritive source. This may explain the longer larvae consumption time (5.5 min) observed in our study, which exceeded the values reported by Fiorilla et al. [17] for Bianca di Saluzzo chickens consuming 5% dry larvae (2.3 min), an indigenous breed that likely possess higher exploratory behavior than commercial breeds [21].

Furthermore, the significant increase in willingness to approach the novel object observed in the ALTER + BSF group indicates that feeding dry BSF larvae stimulates exploratory behavior and long-term habituation compared to both standard and alternative plant-based diets. This aligns with the study of Tahamtani et al. [18], who reported that feeding dried BSF exerted less fear in the novel object test. Star et al. [12] reported a similar effect with the gradual provision of live BSF to older laying hens, which stimulated expression of investigation behaviors. The non-significant differences between the dietary groups regarding the avoidance distance test suggest that the use of alternative ingredients or dried BSF larvae did not increase fearfulness towards humans.

The body condition assessment results revealed dirtier plumage in the CONTROL group, which suggests that excreta of hens in this group were more wet or more viscous than that in the ALTER group. This may be explained by the increased fiber in the alternative diets, which affects excreta moisture and litter dryness, leading to reduced soiling, contact dermatitis risks and ammonia excretion [32]. Kalmendal et al. [33] showed that broilers fed a high-fiber diet with roughage had better plumage quality, cleaner feathers and better hock burn scores compared to birds fed standard diet. Patt et al. [34] reported that hens fed diets with higher insoluble fiber showed significantly better plumage quality over time. Furthermore, hens fed ALTER + BSF diet showed plumage cleanliness scores similar to those of the ALTER group, suggesting that supplementation with dried BSF larvae did not have a negative effect in this regard.

Comb pecking wound scores showed that hens fed ALTER diet experienced social aggression as a stress response at the beginning of the trial. This behavior was likely driven by frustration associated with the novel feed ingredients, whereas the CONTROL group showed minimal aggression. Interestingly, hens fed ALTER + BSF diet exhibited intermediate pecking scores in the beginning, suggesting that dried BSF larvae partially attenuated this stress, likely due to the high palatability of BSF [35] or its effect as environmental enrichment [11], thereby facilitating better acclimation compared to the ALTER diet. However, these differences were limited to the beginning of the trial, with no major adverse effects on feed intake or laying performance observed. Thereafter, pecking comb scores declined, reflecting restored social stability over the trial. Consistently, the unaffected feather condition scores and the lack of plumage damage in the back of the head indicate that the dietary treatments did not induce stress related to feeding insufficiency [36].

When observing the hen’s overall behavioral profile, dietary treatment has a limited impact. In contrast, a detailed temporal analysis revealed clear and significant dietary effects. The decreased grooming activity observed in the ALTER group during the morning reflects a negative impact of this diet on the comfort of the hens. However, feeding ALTER + BSF diet effectively maintained morning grooming durations to levels close to those of the CONTROL group, suggesting that dried BSF larvae contributed to attenuating the stress associated with the alternative diet, which aligns with their effects on the comb condition state observed at the start of the trial.

Furthermore, afternoon observations showed that ALTER + BSF diet actively promoted positive behaviors. Hens in this group showed a significant increase in dustbathing activity compared to the other groups, where this behavior was rarely observed. Likewise, they spent significantly more time sitting in the afternoon, indicating that they were calmer and resting more effectively, likely due to satiety or post-eating satisfaction.

Notably, the provision of dried BSF larvae shifted the pooled time budget of the hens away from passive behaviors toward eating during BSF distribution times. A significant effect of dietary treatment on eating activity was observed in the afternoon of day 42, when hens in the ALTER + BSF group exhibited significantly lower eating levels compared to the other groups. Thereafter, by the end of the trial, hens receiving BSF larvae spent significantly less time standing because they were engaged in BSF eating, whereas the CONTROL and ALTER groups spent more time standing inactive during BSF distribution. This preference for BSF larvae eating over passive behaviors (standing, sitting) supports its status as a high-value reward for hens. Notably, this high motivation was not associated with an increase in aggressive behaviors, indicating the absence of resource-guarding or social stress signs. Although research on the behavioral effects of dried BSF larvae is currently limited, existing studies have reported positive effects. For instance, Tahamtani et al. [18] found that using dried BSF larvae as environmental enrichment for commercially housed laying hens increases foraging time and comfort behaviors, such as wing flapping, tail wagging and preening, while reducing feather pecking. Unlike Ipema et al. [11], who found that scattering dried BSF larvae promotes foraging and walking, our results showed that feeding dried BSF larvae on plates reduced walking and had no effect on foraging. This is likely because plate presentation made the larvae immediately accessible and clearly visible, allowing hens to engage directly in eating, bypassing the search phase [37].

Furthermore, although all groups showed similar eating time budgets at the beginning and middle of the trial, the introduction of plates elicited marked feeding-related excitement across all treatments, reflecting anticipatory behavior toward a food-predictive stimulus [38]. By the end of the trial, hens in the CONTROL and ALTER groups reduced their feeding activity when repeatedly exposed to empty plates, resulting in an extinction of the feeding response rather than a satiation effect [39]. In contrast, hens receiving dried BSF larvae showed high feeding motivation throughout the trial. This sustained responsiveness indicates that their behavior was maintained by the consummatory and reinforcing properties of the larvae, confirming that the observed feeding activity was reward driven [40].

## 5. Conclusions

Although the sustainable diet reduced certain comfort behaviors, it improved plumage condition without negatively affecting fear or exploration. The hens showed increasing interest in consuming the larvae with enhanced adaptability to locally sourced feedstuffs, reflected by promoted exploring and accelerated habituation to novelty. BSF larvae also supported positive afternoon welfare behaviors, with sitting behavior being further promoted and dustbathing events emerging in the BSF-supplemented group.

Overall, topping with dried BSF larvae appears to be an effective environmental enrichment practice, facilitating the habituation of laying hens to locally sourced diets in rural farming systems.

## Figures and Tables

**Table 1:** Ingredients (%) and nutrient composition of the CONTROL and ALTER diets.

	CONTROL	ALTER
	(%)	
Corn	62.6	35.2
Soybean meal	26.2	17.3
Triticale	0	20
Faba beans	0	10
Rapeseed meal	0	5
Soybean oil	0	1.35
Salt	0.35	0.37
Dicalcium phosphate	1.5	1.39
Limestone	8.97	9
Methionine	0.08	0.09
PREMIX ^1^	0.3	0.3
BSF dried larvae	0	0
Calculated analysis		
AME (MJ/kg)	11.44	11.44
CP	17.01	17.07
Methionine	0.39	0.38
Methionine + Cysteine	0.69	0.69
Lysine	0.91	0.9
Tryptophan	0.19	0.19
Threonine	0.66	0.64
Available P	0.34	0.35
Na	0.15	0.16
Cl	0.25	0.27
Vitamin K (mg/kg)	0.76	0.76
Vitamin A (kIU)	7.2	7.2
Vitamin D (kIU)	1.98	1.98
Vitamin E (mg/kg)	6.57	6.57
Laboratory analysis (%)		
Dry matter	89.1	89.2
Organic matter	88.3	89.1
Starch	42.6	41
Crude fat	2.3	2.3
Neutral detergent fiber (NDF)	7.09	7.6
Acid detergent fiber (ADF)	3.07	2.22

^1^ PREMIX: The vitamin and mineral premix contained the following components per kilogram of premix: calcium: 18.89%, sulfur: 3.87%, chlorine: 2.90%, magnesium: 0.35%, methionine: 16.96%, methionine + cysteine: 16.96%, manganese: 34.392 g/kg, zinc: 27.7 g/kg, iron: 24.628 g/kg, copper: 2.754 g/kg, iodine: 640 mg/kg, cobalt: 133.40 mg/kg, selenium: 104.04 mg/kg, choline: 114.8 g/kg, vitamin PP (Niacin): 5.4 g/kg, vitamin B5: 2700 mg/kg, vitamin B2: 1350 mg/kg, vitamin B6: 675 mg/kg, Vitamin B1: 448.34 mg/kg, vitamin K3: 360.07 mg/kg, folic acid: 90.9 mg/kg, vitamin B12: 2.7 mg/kg, Vitamin A: 3599 kIU, vitamin E: 3284.10 IU, vitamin D3: 989.89 kIU.

**Table 2:** Scoring criteria used for the assessment of laying hen welfare and physical condition.

Parameter	Scores
Plumage cleanliness	0 = plumage completely clean and dry 1 = small, localized spots of dirt or fecal material 2 = moderately dirty feathers with noticeable dirt or fecal contamination on one or more body regions. Plumage may appear clumped or slightly matted. 3 = severely dirty feathers. Extensive dirt or fecal contamination covers large areas of plumage. Feathers heavily matted or stuck together.
foot pad dermatitis	0 = feet intact 1 = minimal proliferation of epithelium 2 = necrosis or proliferation of epithelium or chronic bumble foot with no or moderate swelling, not dorsally visible 3 = swollen (dorsally visible)
Hock burn	0 = skin covering the hock joint is intact and smooth 1 = superficial changes affecting small, localized areas with slight reddening or light brown staining). No swelling detected. 2 = clear and visible lesions on the hock joint with dark discoloration, thickened skin, scrabs or crusts. Possible ulceration on open sores may be associated with inflammation or swelling.
Feather condition for back, neck, rump and belly	0 = completely feathered (only single feathers lacking) 1 = feather intact but ruffled or one or more featherless areas < 5 cm in diameter at the largest extent 2 = at least one featherless area ≥ 5 cm in diameter at the largest extent
Plumage damage in the back of the head	0 = complete feathering 1 = feather intact but ruffled or one or more featherless areas < 5 cm in diameter at the largest extent 2 = at least one featherless area ≥ 5 cm in diameter at the largest extent
Skin lesion	0 = no lesions, less than 3 pecks (punctiform damage < 0.5 cm of diameter) or scratches. 1 = at least one lesion ≥ 0.5 cm < 2 cm of diameter at largest extent or ≥3 pecks or scratches 2 = at least one lesion ≥ 2 cm of diameter at largest extent
Comb pecking wounds	0 = no evidence of pecking wounds 1 = less than 3 pecking wounds 2 = 3 pecking wounds and more.

**Table 3:** Ethogram of natural behaviors under observation.

Behavior	Description
Positive affective states [24]	
Dustbathing	Laying down in substrate, head rubbing, and shaking wings vertically, and/or raking the substrate closer to the body with their beak.
Foraging	Scratching and pecking at the ground from a standing, sitting, or walking position.
Grooming	Running beak through feathers in a seated or standing position. No aspects of dustbathing behavior observed.
Floor pecking	Pecking the ground while walking or standing
Eating	Bird is standing with its head over the feeder (or plate for the hens receiving BSF)
Drinking	Bird is standing or sitting and pecking at drinker
Wing flapping	Wing extension and vigorous shaking
Agonistic and damaging behaviors [25]	
Severe feather pecking	Forceful manipulation or removal of other bird’s feathers
Aggressive pecking	Pecking on head, fighting, sparring
Gentle pecking feather	Light pecking other bird’s feather without causing damage
Maintenance behaviors [26]	
Standing	Not moving on two feet, body not touching the floor
Sitting	Body and both hocks touching the floor underneath or directly on either side of the bird
Walking	Locomotion, the first foot is put down on the floor before the second one is lifted (without pecking or scratching)

**Table 4:** Approach frequency (LS means) to a novel object in laying hens fed standard (CONTROL), alternative without (ALTER) or with black soldier fly supplementation (ALTER + BSF) diets (*n* = 5), assessed on four test days during the trial.

	CONTROL	ALTER	ALTER + BSF	Pooled S.E.M	Diet		Day		Diet × Day	
	LS Means (%)				χ^2^	*p*	χ^2^	*p*	χ^2^	*p*
Day 10	35.6 a	24.8 b	26.9 ab	2.8						
Day 30	18.7 b	28.0 a	33.0 a	2.8	14.78	<0.001	72.12	<0.001	102.75	<0.001
Day 47	17.5 b	25.5 b	41.7 a	2.7						
Day 63	19.3 b	27.5 b	38.5 a	2.7						
Overall	22.7 b	26.4 b	34.8 a	2.3						

Different letters (a, b) indicate significant differences between the groups (*p* < 0.05).

## Data Availability

Data are available upon request from the corresponding author.
